# Microfluidic Microwave Sensor for Detecting Saline in Biological Range

**DOI:** 10.3390/s19040819

**Published:** 2019-02-17

**Authors:** Joni Kilpijärvi, Niina Halonen, Jari Juuti, Jari Hannu

**Affiliations:** Microelectronics Research Unit, Faculty of Information Technology and Electrical Engineering, University of Oulu, P.O. Box 4500, FI-90014 Oulu, Finland; niina.halonen@oulu.fi (N.H.); jajuu@ee.oulu.fi (J.J.); jari.hannu@oulu.fi (J.H.)

**Keywords:** lab-on-a-chip, RF sensor, microfluidic sensing, IDE

## Abstract

A device for measuring biological small volume liquid samples in real time is appealing. One way to achieve this is by using a microwave sensor based on reflection measurement. A prototype sensor was manufactured from low cost printed circuit board (PCB) combined with a microfluidic channel made of polymethylsiloxane (PDMS). Such a sensor was simulated, manufactured, and tested including a vacuum powered sample delivery system with robust fluidic ports. The sensor had a broad frequency band from 150 kHz to 6 GHz with three resonance frequencies applied in sensing. As a proof of concept, the sensor was able to detect a NaCl content of 125 to 155 mmol in water, which is the typical concentration in healthy human blood plasma.

## 1. Introduction

Microwave sensors are defined as sensors that transmit an electromagnetic wave (typically between 300 MHz–300 GHz) through the sample or allow a measuring wave to be reflected from the sample and acquire data in the form of scattering parameters (S-parameters), which include wave magnitude and phase information. A wide frequency span is desirable due to the fact that all materials have unique properties in different parts of the electromagnetic spectra, but a common limitation is that the sensor becomes too expensive at a higher frequency. The practical difference between a transmission and reflection sensor is that the latter needs only an input (1-port) while the former needs both an input and an output (2-port) [1]. Two-port measurements are more precise, but also add complexity (design, calibration, and more wiring) to the measurement system.

A typical microwave sensor that employs the 1-port measurement principle uses a coaxial line sensor structure. This is a powerful method which includes mathematical formulae for calculating the dielectric properties of samples [2,3]. Commercial sensors using this principle are available, such as the Speag DAK (Dielectric Assessment Kit) [4]. A coaxial sensor can have 2D or 3D geometry and may be inserted in the sample (e.g., in the case of a liquid) or the sample may be brought into contact with the sensor [3]. In some applications it is preferable to use some other probe geometry such as a split ring resonator (SRR) or interdigitated electrode (IDE). The sensing electrode can then be integrated on basic electronic materials/substrates like printed circuit board (PCB) or ceramic (e.g., low temperature co-fired ceramic, LTCC), although this can make the design of the sensor difficult. Fortunately, there is electromagnetic simulation software available which can be used as a powerful tool for designing custom probes for specific tasks [5].

With a typical commercial sensor such as the Speag DAK it is recommended to have at least 100 mL of liquid sample in order to perform accurate measurements. Homogenous samples can be difficult to obtain in the case of rapidly occurring segregation or sedimentation of particles mixed with fluid. For smaller volumes and expensive samples, microfluidics are nowadays a typical method to handle the samples [6]. By combining a microwave sensor with a microfluidic setup milliliter to nanolitre sample volumes can be used. A wide range of microwave sensing techniques have been used: bare waveguide (coplanar (CPW) and substrate integrated (SIW)) [7,8,9], IDE [5,10], cavity [11,12,13], hairpin [14], split ring [15,16], and special planar [11,17,18,19,20] resonators. Moreover, monitoring of small droplets is presented in [21].

In this paper, we focus on 1-port measurement (S11) of liquid samples. The benefit of the method is that only one cable is needed to connect the sensor. This allows the use of a simple and compact 1-port vector network analyzer (VNA) and provides potential for further miniaturization of the proposed sensor. This kind of sensor structure has been used to measure, for example, food aging [20], cells [5], and chemicals [11]. This paper presents an inexpensive solution that combines a microwave sensor with microfluidics to measure small volumes of biological liquids by using a vacuum driven flow. Also, the sensor itself could be connected directly to a VNA through a cable and SMA connector, so there was no need for cumbersome probe connections. Moreover, the fluidic inlet and outlet were robust and it was easy to connect the µm-scale channel to the mm scale tubing.

The first part of the paper describes the simulation and manufacturing of the sensor. The second part is about sensor characterization using water isopropanol mixtures and the chosen samples were related to human blood plasma, which has a specific salinity. If the salinity is not correct in blood plasma it can cause severe health problems [22]. By using saline water described as blood phantom, we characterized the sensor and established a baseline for future biological samples. The measured data could be seen in real-time providing crucial information about phenomena on the sensor surface.

## 2. Materials and Methods

### 2.1. Sensor Design

**Figure 1:**
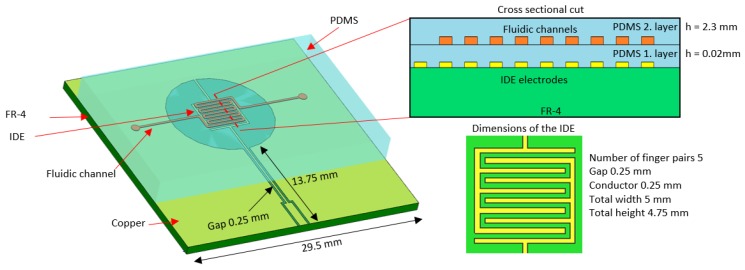
Schematic of the sensor (cross sectional cut is not in scale).

Using electromagnetic simulation software (CST Microwave Studio), the sensor was designed to have several structural resonances when a liquid sample was present. The chosen materials were FR-4 printed circuit board (PCB) and PDMS (polydimethylsiloxane) for the microfluidics, which has been proven to work successfully with PCB [23]. The actual sensing part was an interdigitated electrode (IDE) which had dimensions related to the microfluidic channel. The channel was designed to have specific dimensions (width of 250 µm and height of 60 µm) due to the manufacturing technique. The IDE structure was chosen because it enabled tuning of the structural resonances to the measured band using a small footprint. The benefit of IDE compared to other resonance structures (e.g., SRR) is that it provides multiple resonances in a relatively narrow band. A schematic of the sensor is shown on Figure 1. The sensor was tested with water-isopropanol solutions with different concentrations and by detecting saline concentration within the biological range using µl volume samples. In the blood plasma of a healthy adult the salinity ranges from 125 to 145 mmol [24,25] and similar amounts were used in this work. The sensor was designed for measurements using a one-port vector network analyzer which had a frequency span from 150 kHz to 6 GHz. A wide frequency range is beneficial for detecting different kinds of sample because all substances, such as human blood, have characteristic dielectric properties which can be referred to as their “electronic fingerprint”. The change in resonance frequency is due changes in the relative permittivity and the attenuation is related to the material’s dielectric losses. Unwanted effects are radiation to surrounding space causing attenuation and unexpected reflections from material boundaries, especially with conductive samples.

### 2.2. Modeling and Simulation

**Figure 2:**
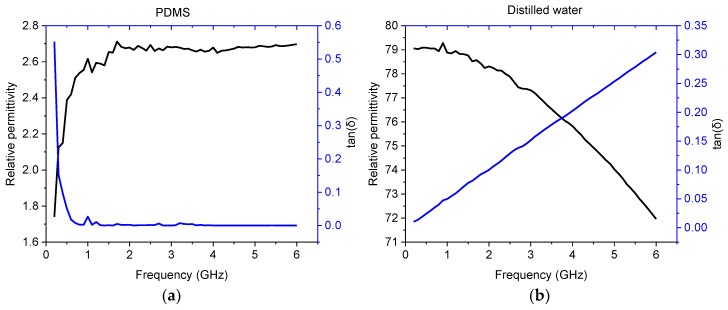
Dielectric properties of the materials measured with Dielectric Assessment Kit (DAK) and used in model: (**a**) polymethylsiloxane (PDMS); (**b**) distilled water.

**Figure 3:**
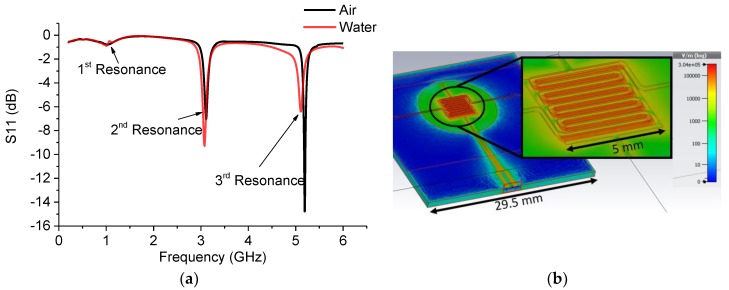
(**a**) Simulated response of the sensor; (**b**) simulation model showing strong electric field coupling on the interdigitated electrode (IDE) area, PDMS top layer is not shown here.

The sensor was modeled using the CST studio and incorporating the dielectric properties of the materials as measured with a Speag DAK-3.5. In Figure 2, the measured relative permittivity and loss tangent are shown as a function of frequency and the FR-4 was modeled with a constant permittivity of 4.5 and a tan δ ~0.05. The simulation was run by the frequency domain solver using open boundary conditions at all borders. Automatic mesh adaptation was used to form a sufficient tetrahedral mesh and then the mesh was locked to give over 2.8 million cells for the following simulation runs. A waveguide port was used to define an edge mounted SMA. The goal of the simulation was to achieve several resonance peaks in the measured band, especially when a water-based sample was present. With the selected design, three resonance peaks were achieved, providing sufficient tools to differentiate targeted analytes. The main restrictions on the dimensions and design were set by the manufacturing techniques which are described in detail in Chapter 2.3. The results and the simulation model are shown in Figure 3 with the resonance frequencies in Table 1.

### 2.3. Manufacturing the Sensor and Microfluidics

The sensor was manufactured from printed circuit board (FR-4, 1 mm/18 µm copper). The PCB was cut and holes were drilled for the fluidic connections using a CNC router (Roland SRM-20), and aligning marks were made on the corners. Laser structuring (LPKF ProtoLaser U3) was used to make the interdigitated electrode structure (IDE) and the transmission line on the PCB using the aligning marks.

**Figure 4:**
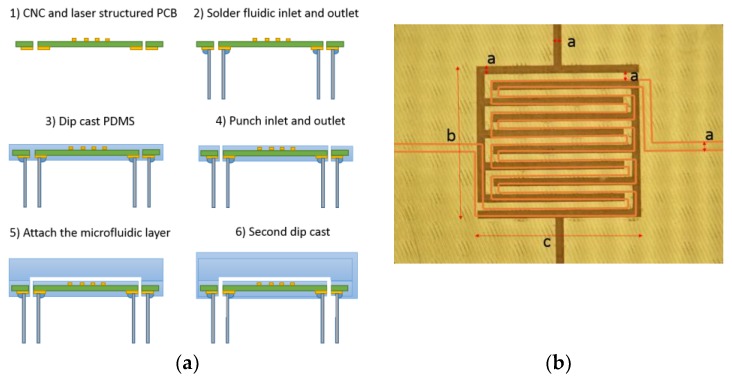
(**a**) Sensor processing steps; (**b**) sensor dimensions (a = 0.25, b = 4.75, and c = 5 mm) and highlighted fluidic channel as orange line.

The fluidic inlet, outlet, and a female SMA connector were attached to the substrate by soldering. Fluidic channels were manufactured by using a combination of mold replication and dip coating techniques with PDMS (Sylgard 184 Silicone Elastomer, Dow Corning). The mold was manufactured by using a microscope glass slide and Kapton tape. The tape was attached to the glass and then laser cut into precise shape to form the mold for the fluidic channel. Degassed PDMS was poured on top of the glass/Kapton mold and cured for 1 h at 100 °C. The dip coating thickness was 20 µm and the height of the replicated top part of the mold was 2.3 mm. The microfluidic channel width was 250 µm and its height was 60 µm, yielding a total volume of 1 µl for the channel. The inlet and outlet were partly filled with PDMS during the dip coating process and were punched with a needle after curing. The top fluidic layer was attached on top of the dip coated IDE and then the inlet and the outlet were plugged using a loop of silicone tubing. The whole structure was dipped in PDMS for a second time and was then cured (1 h at 100 °C) yielding a robust structure. The silicone tubing was removed before curing to prevent pressure build up which could cause delamination. Figure 4 shows the processing steps and sensor dimensions.

Finally, Teflon tubing (1 mm inner diameter) was attached to the fluidic connectors using a short silicone tubing sleeve and the VNA was connected to the sensor via an SMA connector.

### 2.4. Sample Loading and Bubbles

**Figure 5:**
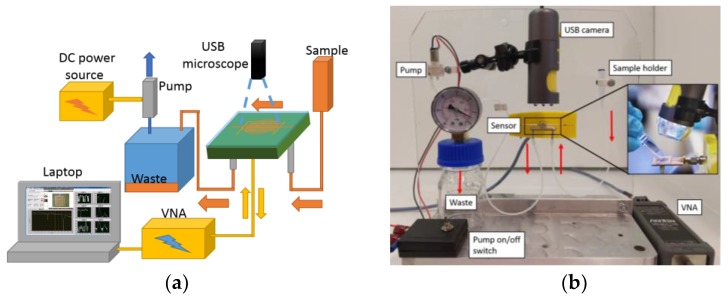
Measurement and sample pumping system: (**a**) schematic presentation (**b**) photograph of the system (the computer and voltage source are not shown).

Sample loading was performed by the flow generated with a voltage-controlled vacuum pump (DC 3V Micro Vacuum Air Pump). The liquid sample was pipetted onto the sample holder and a vacuum was generated at the output of the sensor using the pump and a glass flask with a manometer. The glass flask contributed to a steady flow as it evened out variations in the pump speed. The samples ended in the glass flask after going through the sensor. Figure 5 shows the sample loading system. Real time video feeds from the experiments were recorded using an optical USB microscope (AVEN Mighty Scope) connected to a computer.

**Figure 6:**
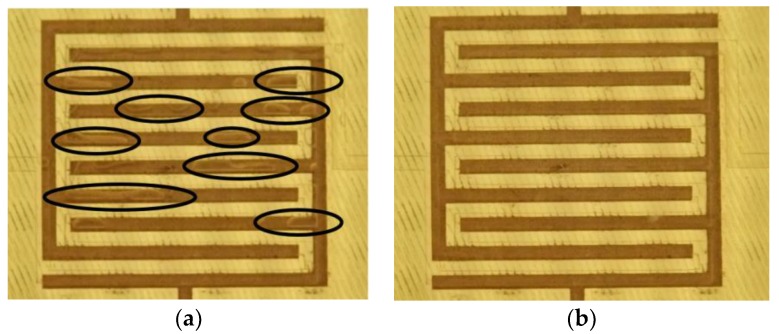
(**a**) Direct sample loading without isopropanol flush showing several bubbles highlighted with black circles; (**b**) sample loading done after isopropanol flush.

Bubbles in the microfluidic channel were a clear problem, especially when changing samples. The problem was solved by using two techniques: The channel was designed so that bubbles could not settle due the high fluid flow in the narrow channels. Secondly, it was solved by using a sample loading process involving an isopropanol flush. First, isopropanol was flushed through the sensor to remove traces of the previous sample and any trapped bubbles. Then the actual sample was pumped into the sensor and pumping was stopped, after which at least 10 measurement cycles were saved. Figure 6 shows comparison of the measurements with and without using an isopropanol flush. The camera was used to observe that there were no bubbles present during the measurements.

### 2.5. Measurement Method

**Figure 7:**
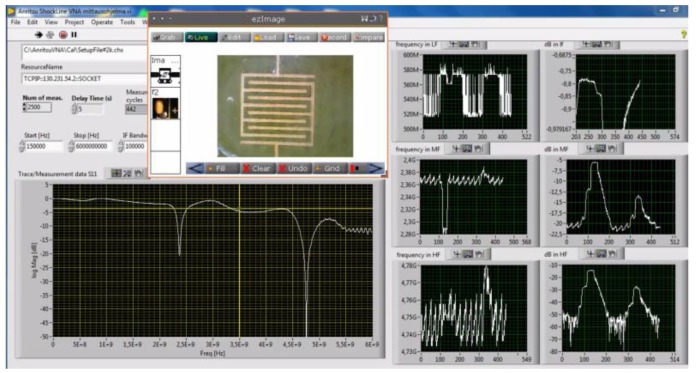
Overview of the LabView user interface. Settings are shown in upper left corner next to the USB real-time microscope image; below is shown the S11 (dB) measurement, and on the right there are three resonance peaks plotted as frequency or attenuation vs. time.

Microwave measurements were performed using a one-port vector network analyzer (Anritsu MS46121B) and the instrument was controlled automatically with tailored LabView software. S11 data over the whole spectrum were saved at 10 s intervals. Moreover, peak data (attenuation minimum and frequency) were saved and plotted in real time. The frequency sweep was from 150 kHz to 6 GHz with 1993 points. Before running the experiments, the VNA was calibrated using open-short-load calibration. Figure 7 shows the software user interface.

### 2.6. Measured Samples

Samples having salinity concentration within the biological range were made using distilled water and sodium chloride (Sigma Aldrich, purity ≥ 99%). The stock solution was diluted to make samples with 125, 135, 145, and 155 mmol saline concentrations. Moreover, the sensor’s response to the permittivity change was tested with different isopropanol-water solutions with isopropanol volume concentrations of 0%, 20%, 40%, 60%, 80%, and 100%. All samples were measured with the proposed sensor and reference measurements were done with a commercial dielectric assessment kit (Speag DAK).

## 3. Results

### 3.1. Simulation Results

**Figure 8:**
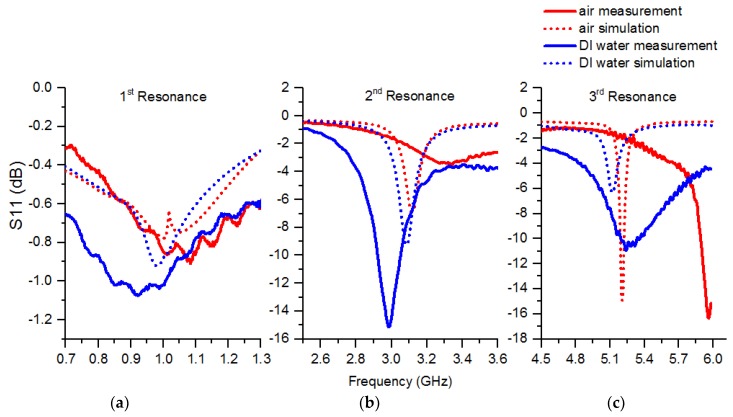
Simulation vs. measurement: (**a**) 1st resonance; (**b**) 2nd resonance; (**c**) 3rd resonance.

Compared to the simulation, the measurements showed a larger variation in frequency at all three resonances. The simulation and measured results with air and pure water are shown in Figure 8, and Table 2 includes information about all three resonances and the total change between the air and water samples.

In the simulation, the first resonance of the sensor showed a shift in resonance frequency to lower frequencies when water was applied in the channel in contrast to air, due the higher relative permittivity. Moreover, there was more attenuation due to the losses presented by water. Measured values showed similar behavior but the shift of the resonance peaks was more pronounced and there was a clear discrepancy between simulation and measurement in the case of water. The second resonance showed a similar trend but the change was more clear in both simulated and measured values.

**Figure 9:**
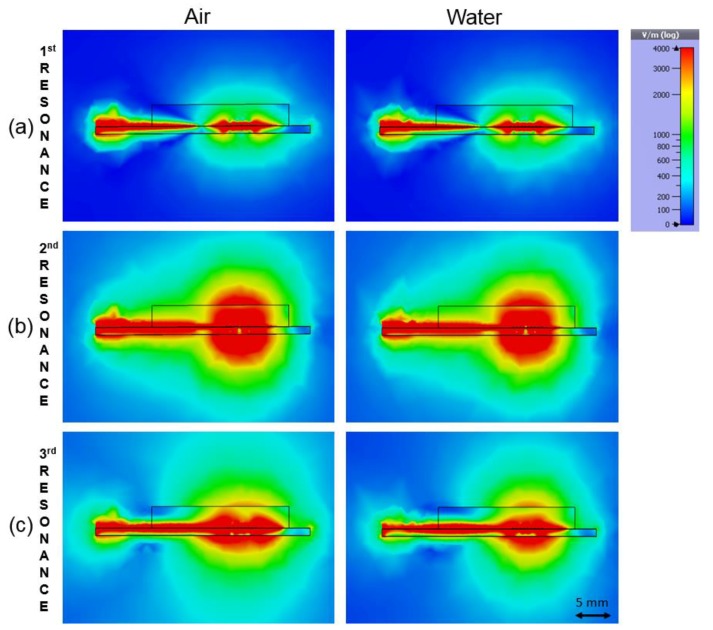
Simulated radiation patterns in three different resonances: (**a**) 1st resonance with air and with water; (**b**) 2nd resonance; (**c**) 3rd resonance where air shows higher radiation to surrounding space.

In the third resonance the shift of the peak was clear but the attenuation behavior was inverted compared to the previous resonances. This can be explained by simulation, where the water is reflecting the electromagnetic wave more than air causing a lower attenuation. With air, there is more radiation to the surrounding space resulting in higher attenuation, as can be seen by comparing the radiation patterns in Figure 9a–c. It can be seen that in the third resonance (Figure 9c) the radiation change is clearly visible compared to Figure 9a,b. Additionally, image analysis made in Figure 9c confirmed the increased radiation. By drawing a closed polygon (threshold 400 v/m) and calculating the area using AutoCAD, the area was estimated to be about 30% larger with air compared to water. These results show that all three resonances need to be inspected individually when interpreting the results due to the different behavior of the dielectric properties of the sample at different frequencies.

The measurement results followed the simulation as expected showing three resonance peaks in the measured band when a water sample was present.

### 3.2. Measurement Results

#### 3.2.1. Isopropanol

**Figure 10:**
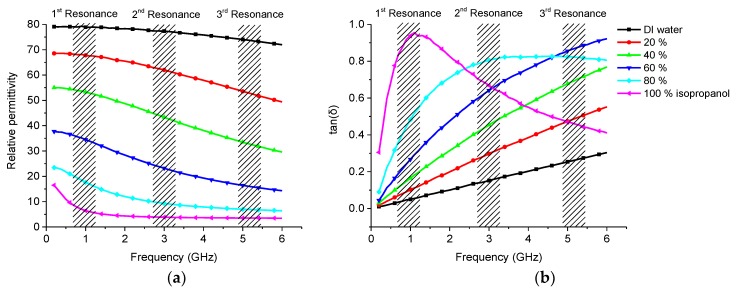
Dielectric properties of the isopropanol-water mixtures measured with DAK: (**a**) permittivity; (**b**) loss tangent.

**Figure 11:**
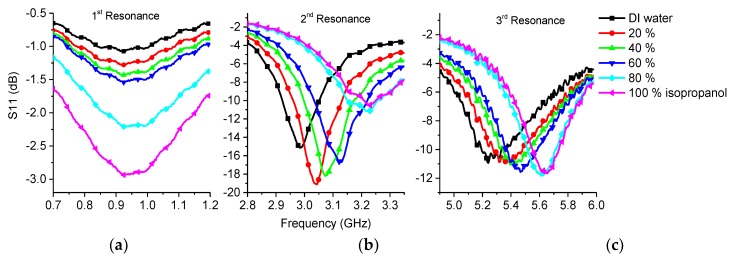
Measurement results of isopropanol-water mixture: (**a**) 1st resonance; (**b**) 2nd resonance; (**c**) 3rd resonance.

Distilled water samples with different volume concentrations of isopropanol were measured using the manufactured sensor. Reference measurements were made with the commercial dielectric assessment kit. Reference measurements are shown in Figure 10 and sensor measurements in Figure 11.

The reference measurements showed that the relative permittivity decreased when the isopropanol content was higher. Losses showed an upward trend with higher isopropanol content, but at around 2.5 GHz the 100% sample had similar losses to those of the 80% sample and they continued to decline with higher frequencies. The 80% solution showed similar behavior but the decline was not so steep with higher frequencies.

In the sensor measurement, the first resonance (Figure 11a) showed higher attenuation as the isopropanol content rose which was caused mainly by a higher loss factor (Figure 10b). Moreover, there was a slight shift towards higher frequencies, which was caused by the lower effective permittivity.

Similar results were seen on second resonance (Figure 11b) and the frequency shift was even more obvious. However, the loss tangent was not logical because the isopropanol content increases made the results unusable for sensing the loss of the sample probably due to the signal radiation to surrounding space, see Figure 10b.

At the third resonance frequency, it could be seen that the permittivity was decreasing when the isopropanol content was higher and the frequency shifted towards higher frequencies as expected. Again, due to the radiation the losses did not match with the reference measurement.

#### 3.2.2. Saline

**Figure 12:**
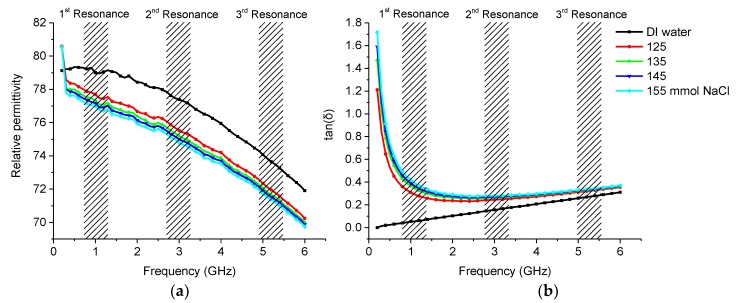
Permittivity and losses of saline water measured with DAK system: (**a**) Permittivity; (**b**) Loss tangent.

DAK measurements showed that the relative permittivity was lower with saline water compared to distilled water. Results can be seen in Figure 12a. However, a higher permittivity at a frequency of 200 MHz was also seen due to the ions having time to cause polarization of the liquid medium and thus causing a higher relative permittivity. Saline in water causes higher losses compared to pure water due to the increasing conductivity as there are free ions present. Ions cause absorption of the electromagnetic wave, which can be seen clearly on the DAK measurements shown in Figure 12b, but this phenomenon disappears at higher frequencies.

**Figure 13:**
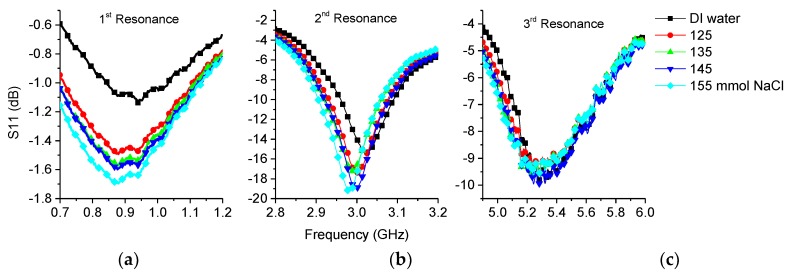
Measurement results of saline water with different concentrations: (**a**) 1st resonance; (**b**) 2nd resonance; (**c**) 3rd resonance.

The sensor clearly showed higher attenuation on the first resonance peak, see Figure 13a, as the loss tan increased with the salinity. The second frequency, see Figure 13b, also showed higher attenuation as the salinity increased. At higher frequency, see Figure 13c, the response was not observed clearly due the small changes in the losses between samples with different salt concentrations.

It should also be noted that the first and second resonances drifted slightly towards lower frequencies despite the permittivity having decreased according to the DAK measurement. This could be caused by the structure where the conductive saline media was between two PDMS dielectric non conducting regions. This could have induced currents in the liquid causing polarization leading to a higher effective permittivity. This phenomenon is not currently well understood and needs further study.

### 3.3. Sensor Performance

**Figure 14:**
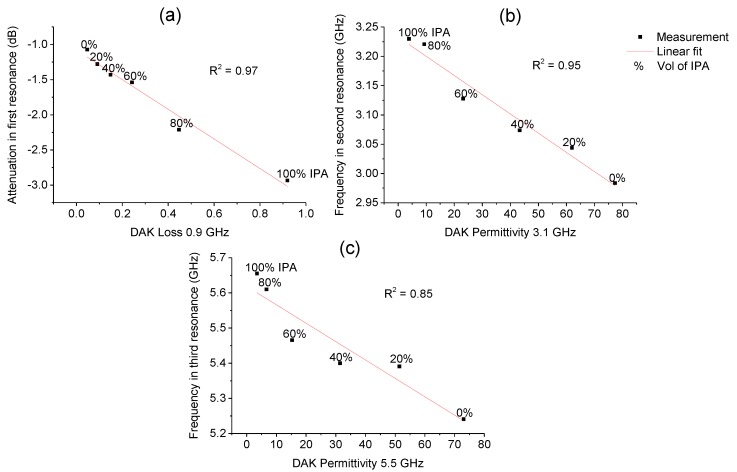
Isopropanol measurements versus DAK measurements: (**a**) first resonance vs. loss tangent; (**b**) second resonance vs. relative permittivity; (**c**) third resonance vs. relative permittivity.

The sensor was characterized by using isopropanol-water samples and showed the best performance on the second resonance and the third resonance in terms of permittivity as a frequency shift. In the case of the first resonance the loss tangent was measured most reliably by inspecting the attenuation. Figure 14 shows the sensor measurement results compared to the reference measurements made with DAK. These data can be used as a linear fit calibration curve for isopropanol measurements.

**Figure 15:**
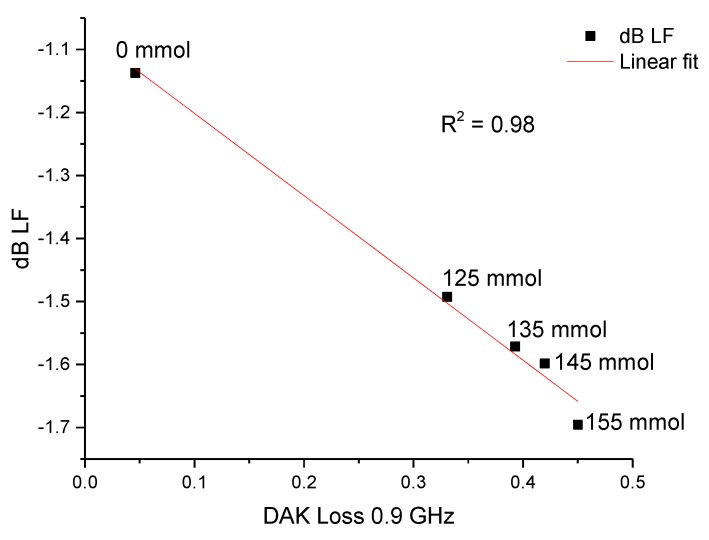
Measurement results of saline water with different concentrations.

With saline samples, a low frequency shows usable results as the attenuation increased as the saline concentration increased. Figure 15 shows the measurement data.

## 4. Discussion

The use of a microwave sensor with an integrated microfluidic system is a versatile and powerful sensing technique. Moreover, the whole measurement system, including the pump, could be used with batteries. This paper shows the proof of concept of such sensors in the case of saline water and water-isopropanol mixtures. The sensor also has potential for measuring liquid samples other than those presented in this paper, as seen in the measurement results of different IPA concentrations with large variations in dielectric properties and also, in contrast, the small changes in dielectric properties seen in saline samples. Analysis of biological samples such as whole blood, urine, and plasma would be an especially interesting aim for further tests.

Comparing the proposed sensor to research mentioned in papers [5,21], which have been published quite recently and are somewhat comparable, it does not need any clean room techniques or substrates, which are typically expensive to process. Also, a similar frequency shift between air and water was observed, indicating that sensitivity was not sacrificed. Moreover, the sensor does not need the impractical probing stage which is needed to connect the VNA. The pumping system was simple and user friendly.

The sensor demonstrated here was comprised of PCB, PDMS, and the sample, which all had different dielectric properties depending on the frequency in use. Moreover, there were several interfaces between the materials due to the IDE and the serpentine fluidic channel. Thus, it was beneficial to measure the dielectric properties separately with accurate laboratory instruments before simulation, thus providing more reliable simulation results.

With VNA in the system as a measuring instrument, sample properties can be measured in a wide frequency band. After determining the relevant parameters at a specific frequency, we can design an electrical measurement system specified for a narrower band which is simpler and cost effective. A PCB substrate is suitable for integrating the necessary electronics. This enables the manufacture of cost effective sensor systems using the presented manufacturing technique. For example, by using an oscillator circuit (e.g., voltage controlled oscillator) and power meter the losses can be measured at a single frequency or a similar system could be implemented by using a frequency meter to measure the frequency change [1,26]. Also, several integrated VNAs (VNA on chip) have been presented in references [27,28,29].

The presented sensor was manufactured without the need for complicated manufacturing techniques. This has some drawbacks in the designing of the sensor due to some dimensions (e.g., the fluidic channel) that cannot be changed freely without compromising structural robustness. However, there is potential for enhancing the sensor. Improving the sensor by adding metal reflectors on the sides and on top of the structure might improve sensitivity by preventing radiation in the surrounding space. Also, fine tuning the dimensions of the IDE and microfluidic channel could be possible without sacrificing reliability.

Interpreting the acquired data was not straight forward due the complex behavior of EM waves on the sensor. All the materials on the top of the sensor affected the measurement, thus the measurement was not only sensing the sample but also the structures around the sample. Also, radiation and reflections from the material interfaces caused uncertainties when measuring relative permittivity and sample loss. These problems with radiation and, in the case of saline water the unexpected shift of resonance to lower frequencies, were not solved completely and need further study. However, the sensor did have clear behavior in specific intervals as shown with saline water and could be used to define salinity.

## 5. Conclusions

In this work, a microwave sensor with a microfluidic system was designed with the aid of EM simulation. The sensor was manufactured and successfully tested with small volumes of isopropanol/distilled water mixtures and saline water with biological concentrations. An easy to use vacuum-powered sample loading system with robust fluidic connections was also designed and built. A user interface for observing real time data was implemented with LabView. Blood phantom samples with saline concentrations from 125 to 155 mmol were measured successfully and the sensor concept is ready for the next generation version. It should also be noted that the sensor was used to measure the salinity in distilled water but it would be possible to utilize the sensor on liquid samples with a wide range of dielectric properties.

## Figures and Tables

**Table 1:** Corresponding frequencies in the simulation model.

Sample	1st Frequency/Attenuation GHz/dB	2nd Frequency/Attenuation GHz/dB	3rd Frequency/Attenuation GHz/dB
Air	1.024/−0.785	3.106/−7.019	5.189/−14.777
DI water	1.006/−0.901	3.077/−9.285	5.113/−6.400

**Table 2:** Corresponding frequencies in the simulation model vs. the measured frequencies.

Sample	1st Resonance/Attenuation GHz/dB	2nd Resonance/Attenuation GHz/dB	3rd Resonance/Attenuation GHz/dB
Air measurement	1.084/−0.908	3.341/−3.499	5.961/−16.359
Air simulation	1.006/−0.778	3.109/−6.859	5.189/−14.919
Water measurement	0.922/−1.074	2.984/−15.140	5.241/−10.937
Water simulation	0.983/−0.921	3.077/−9.224	5.107/−6.318
Air-water measurement	Δ(−0.162/−0.166)	Δ(−0.357/−11.641)	Δ(−0.720/+5.422)
Air-water simulation	Δ(−0.023/−0.143)	Δ(−0.032/-2.365)	Δ(−0.082/+8.601)
